# FGF2 Induces Resistance to Nilotinib through MAPK Pathway Activation in KIT Mutated Melanoma

**DOI:** 10.3390/cancers12051062

**Published:** 2020-04-25

**Authors:** Pauline Tétu, Julie Delyon, Jocelyne André, Coralie Reger de Moura, Malak Sabbah, Ghanem E Ghanem, Maxime Battistella, Samia Mourah, Céleste Lebbé, Nicolas Dumaz

**Affiliations:** 1 INSERM, U976, Team 1, Human Immunology Pathophysiology & Immunotherapy (HIPI), F-75010 Paris, France; 2 Département de Dermatologie, Hôpital Saint Louis, AP-HP, F-75010 Paris, France; 3 Institut de Recherche Saint Louis (IRSL), Université de Paris, F-75010 Paris, France; 4 Département de Pharmacogénomique, Hôpital Saint Louis, AP-HP, F-75010 Paris, France; 5 Laboratory of Oncology and Experimental Surgery, Institut Jules Bordet, Université Libre de Bruxelles, Rue Héger-Bordet 1, 1000 Brussels, Belgium; 6 Département de Pathologie, Hôpital Saint Louis, AP-HP, F-75010 Paris, France

**Keywords:** melanoma, targeted therapy resistance, KIT, MAPK, FGF2 BIM

## Abstract

KIT is a bona fide oncogene in a subset of melanoma and, ex vivo, KIT inhibitors are very efficient at killing KIT-mutant melanoma cell lines. However, KIT-mutant melanoma tumors tend to show a de novo resistance in most cases and a limited duration of response when response is achieved. We performed pharmacodynamic studies on patients with KIT-mutated melanoma treated with nilotinib, which suggested that the FGF2 axis may be a mechanism of resistance in this subset of melanoma. Using several melanoma cell lines, which are dependent on oncogenic KIT, we showed that although KIT inhibition markedly decreased cell viability in melanoma cell lines with distinct KIT mutations, this effect was lessened in the presence of FGF2 due to inhibition of BIM expression by MAPK pathway activation. Addition of a MEK inhibitor reversed the FGF2-driven resistance for all KIT mutants. We confirmed the expression of FGF2 and activation of MEK-ERK in melanoma patients using in situ data from a clinical trial. Therefore, the combined inhibition of KIT with FGFR or MEK may be a next-step effective clinical strategy in KIT-mutant melanoma.

## 1. Introduction

The receptor tyrosine kinase KIT is well recognized for its importance in melanocyte development [1]. Upon binding of the KIT ligand (also known as stem cell factor, SCF), KIT autophosphorylates its intracellular domain leading to activation of the downstream pathways mitogen activated protein kinase (MAPK), phosphatidylinositol 3-kinase (PI3K), and janus kinase/signal transducers and activators of transcription (JAK/STAT) [2]. KIT plays an essential role in melanocyte growth and survival, at least in part through activation of the melanocyte master regulator microphthalmia-associated transcription factor (MITF) that is known to control critical melanocyte functions [3]. Many studies have reported the incidence of KIT mutations and focal amplifications in melanoma and their association with clinicopathologic characteristics [4]. The Cancer Genome Atlas (TCGA) Network classified cutaneous melanoma into BRAF, NRAS (NRAS proto-oncogene), NF1 (neurofibromin 1), and triple-wild-type groups. About 22% of triple-wild-type melanomas contain KIT aberrations [5]. KIT alterations define a unique subtype of melanoma associated closely with older age, and acral, mucosal, and chronic sun damage sites, but not associated with sex, histological features, or tumor stage [6,7]. Although the KIT mutation rate in melanoma is higher in Caucasian than Asian populations, there is no significant difference in the clinical association with KIT mutations between the two groups [8]. In contrast to BRAF and NRAS mutations, somatic mutations in KIT are distributed throughout the entire gene. However, they are typically found in four hot-spots (L576, K642, W557-V560, and D816-A829) where 70% of KIT mutations in melanoma can be found [9].

We and others have shown that KIT is a bona fide oncogene in a subset of melanoma, driving tumor cell proliferation, progression, and migration through the constitutive activation of downstream signaling cascades, therefore demonstrating that KIT is a therapeutic target in these tumors [10,11]. Early ex vivo studies demonstrated that KIT inhibitors were efficient in killing KIT-mutant melanoma cell lines [12]. However, KIT-mutant melanoma tumors tend to show a de novo resistance in most cases and a limited duration of response when response is achieved [13]. In KIT-mutated gastro intestinal stromal tumors (GIST), treatment with imatinib, a selective inhibitor targeting KIT, ABL, and platelet-derived growth factor receptor (PDGFR), results in an overall response rate of approximately 50% in patients with durable responses and a median progression-free survival of 18 months [14]. In contrast, clinical studies examining the efficacy of KIT inhibitors in patients with KIT-mutated melanoma have been underwhelming compared with GIST. Treatment with KIT inhibitors resulted in a trend towards improved response in melanoma patients, with response rates of approximately 20%. Despite the clinical benefit achieved with KIT inhibition in select patients with melanoma harboring KIT mutations, most patients ultimately experience disease progression [10,15,16,17,18,19]. Understanding the resistance to KIT inhibitors is therefore important to improve knowledge of tumor biology and the design of clinical research protocols with KIT inhibitors. Importantly, mechanisms of secondary resistance in GIST commonly involve the development of secondary KIT mutations affecting the tyrosine kinase domains in exons 13 and 17 [20]. This differs from mechanisms of resistance observed in melanoma driven by KIT alterations where, thus far, no such secondary mutations have been observed. Mechanisms of resistance to KIT inhibitors in melanoma remain unclear. Limited available data suggest that, in melanoma, the development of secondary mutations in NRAS or CTNNB1, overexpression of MET, and activation of the mTOR pathway by alternative mechanisms can confer acquired resistance to imatinib [19,21,22,23]. These mechanisms have only been described in single patient highlighting the need for additional mechanistic studies, molecular profiling, and evaluation of combination therapies. We performed a pharmacodynamic study during a phase II clinical trial with nilotinib in KIT-altered melanoma, and showed that the expression of growth factors, such as FGF2, was significantly high at baseline and reduced during nilotinib therapy in responders. In light of these data, we explored the response of KIT mutated melanoma cell lines to KIT inhibitors under specific cellular conditions and microenvironments. We showed that although KIT inhibition markedly decreased cell viability in melanoma cell lines with distinct KIT mutations, this effect was lessened in the presence of FGF2 due to inhibition of BIM expression by MAPK pathway activation. Addition of a MEK inhibitor reversed the FGF2-driven resistance for all KIT mutants. We hence highlighted a possible mechanism of resistance which can be countered by combination therapy.

## 2. Results

### 2.1. KIT Mutated Melanoma Are Sensitive to All KIT Inhibitors

**Figure 1:**
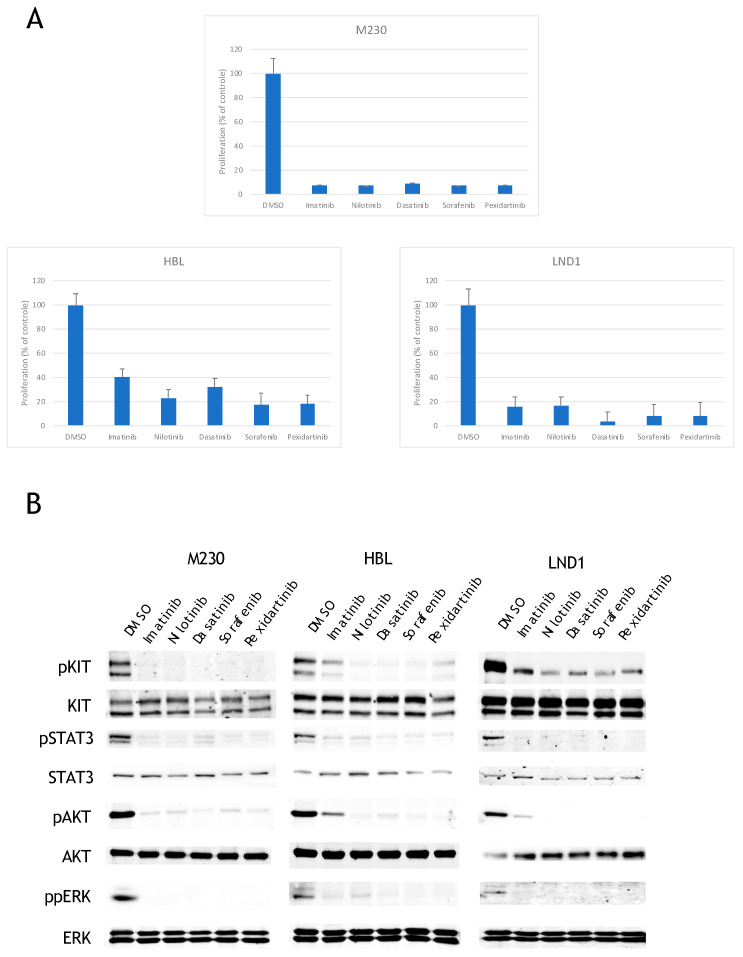
Effects of KIT inhibitors on proliferation and signaling. (**A**) Cells were treated with DMSO or 1 µM of inhibitors and proliferation was analyzed after 3 days (data are represented as mean +/− SD). All inhibitors induced a significant inhibition a proliferation in the three cell lines (*p* < 0.001; unpaired *t*-test). (**B**) Cells were treated for 24 h with DMSO or 1 µM inhibitors and the levels of phosphorylated proteins or total proteins were analyzed by Western blotting.

To better understand the mechanism by which KIT mutated melanoma resist to KIT inhibitors, we used three melanoma cell lines with different KIT alterations: M230 (L576PKIT), HBL (D820YKIT), and LND1 (amplification of WTKIT). We verified the presence of each specific KIT mutation within each cell line using Sanger sequencing and KIT locus amplification using quantitative PCR [24]. No BRAF or NRAS mutations, or secondary mutations in KIT were detected in any of the cell lines. We evaluated expression of KIT at the membrane using FACS analysis and showed that all three cell lines expressed KIT at their membrane with M230 expressing the lowest amount and LND1 expressing the highest amount of receptor at the membrane (Figure S1). To better understand the mechanisms of resistance to single-agent KIT inhibition, we first tested the efficacy of five different KIT inhibitors available in the clinics: imatinib, nilotinib, sorafenib, dasatinib, and pexidartinib. All five inhibitors markedly decreased cell viability of M230 and LND1 with similar efficacy at a dose of 1 µM (Figure 1A). HBL was less sensitive to the inhibitors in general, and especially to imatinib, in agreement with the resistance of the D820Y mutant to imatinib described in Ba/F3 cells [25] (Figure 1A). To determine the endogenous constitutive activity of each KIT mutation, we assessed the phosphorylated levels of KIT in each melanoma cell line by Western blot. We showed that KIT was constitutively phosphorylated in the three melanoma cell lines. KIT phosphorylation was inhibited by all five inhibitors in all the cell lines, although imatinib was less efficient at inhibiting KIT in HBL (Figure 1B). We evaluated activation of the downstream pathways MAPK, PI3K, and JAK/STAT by measuring phosphorylation of ERK, AKT, and STAT3 respectively. All three proteins were phosphorylated in the M230, HBL, and LND1 cell lines, and their phosphorylation was inhibited with the different KIT inhibitors, demonstrating that all three signaling pathways were activated downstream of oncogenic KIT (Figure 1B).

### 2.2. A Link Between FGF2 and Resistance

**Figure 2:**
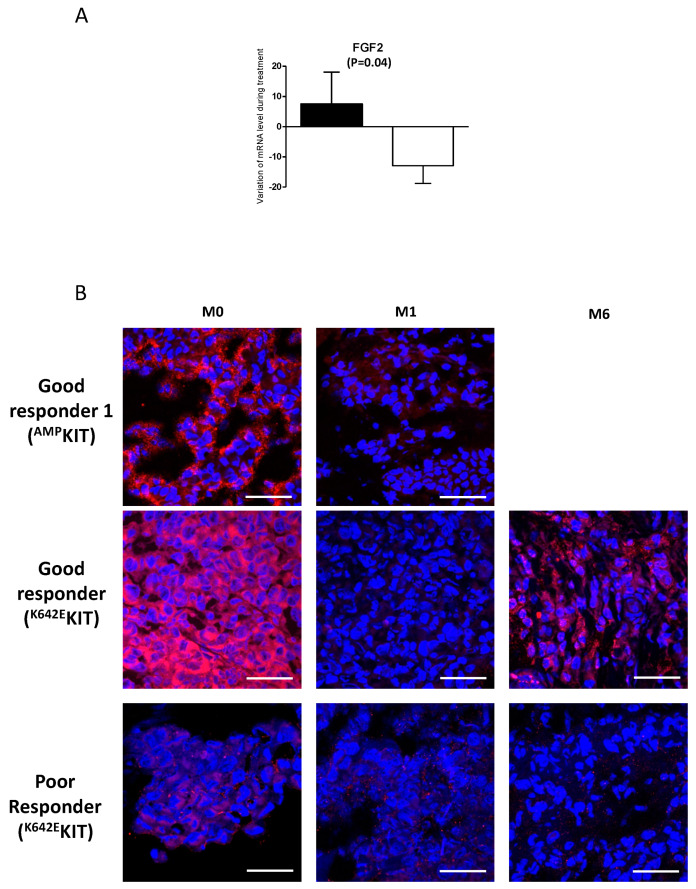
Variation of FGF2 expression during treatment. (**A**) Variation in expression between baseline and after 1 month of treatment with nilotinib of FGF2 mRNA expression, in patients treated with nilotinib with poor (black bar) or good (white bar) response following RECIST (respectively, *n* = 8 and *n* = 5). Box plot: middle bar, median; lower and upper box limits, 25th and 75th percentiles, respectively; whiskers, min and max values. Variables were compared with the Mann–Whitney test one tailed. (**B**) FGF2 expression in tumors assessed by immunofluorescence. Representative photographs of FGF2 stained in red in two good responders and a poor responder at baseline, and after 1 (M1) and 6 months (M6) of treatment. KIT alterations are indicated for each patient (^AMP^KIT = amplification of the KIT locus). DAPI stained cell nuclei (blue). Scale bar, 50 μm.

We previously performed a pharmacodynamic study on 25 patients, using a quantitative PCR array, during a phase II clinical trial with nilotinib in KIT-altered melanoma [16]. Amongst the 25 patients, 13 were classified as poor responders (PD or SD < 3 months, *n* = 8) and five as good responders (*n* = 5). For the patients who had baseline and follow-up tumor samples available, we measured the variation of mRNA level during treatment of 12 growth factors (EGF, FGF1, FGF2, FIGF, HGF, IGF1, PDGFA, PGF, TGFB1, VEGFA, VEGFC, and VEGF121). We noticed a trend towards a reduction of growth factor expression in good responders compared to poor responders, suggesting a link between growth factors and resistance to nilotinib (Figure 2A and data not shown). As FGF2 showed a significative decrease (*p* = 0.04) between poor and good responders to nilotinib, we evaluated its expression by immunofluorescence in available samples from good and poor responders at baseline and follow-up. We showed that FGF2 was strongly expressed in good responders and decreased upon treatment, whereas it was not expressed in poor responders at baseline or after treatment (Figure 2B). Interestingly, one good responder showed a decrease of FGF2 after 1-month treatment followed by an increase after 6-month treatment highlighting a link between FGF2 expression and resistance to nilotinib.

**Figure 3:**
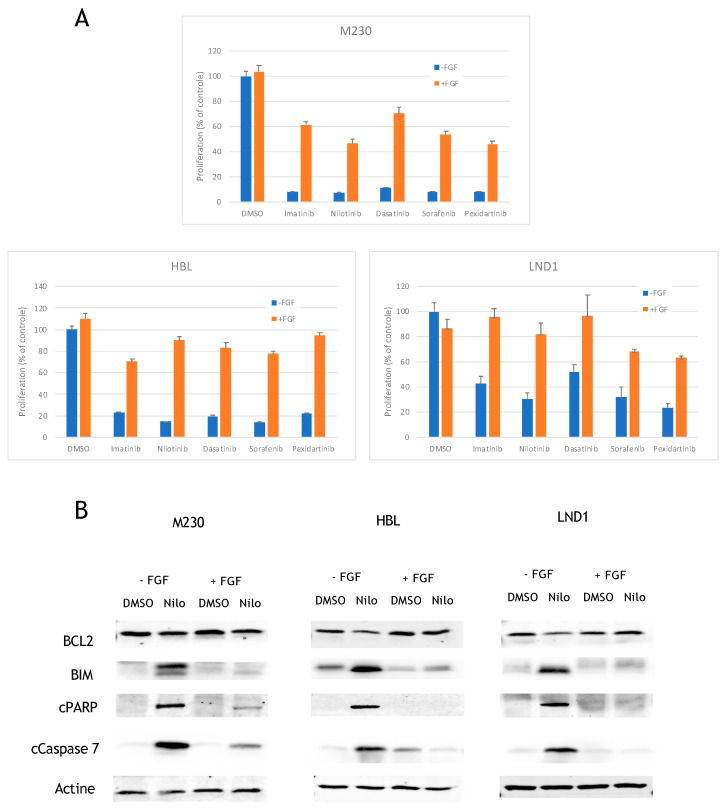
Effects of FGF2 on proliferation and signaling. (**A**) Cells were treated with DMSO or 1 µM of inhibitors in the absence or in the presence of 20 ng/mL FGF2 and proliferation was analyzed after 3 days (data are represented as mean +/− SD). The effect of all KIT inhibitors was significantly reduced in all three cell lines in the presence of FGF2 (M230, *p* < 0.002; HBL, *p* < 0.01; LND1, *p* < 0.02; unpaired *t*-test). (**B**) Cells were treated for 24 h with DMSO or 1 µM nilotinib in the absence or in the presence of 20 ng/mL FGF2 and the levels of cleaved proteins (cPARP and cCaspase 7) or total protein were analyzed by Western blotting.

To confirm this hypothesis, we sought to determine the effect of FGF2 on KIT inhibition by nilotinib ex vivo. M230, HBL, and LND1 cell lines were treated with the five different KIT inhibitors in the absence or in the presence of FGF2. As previously shown, all five inhibitors markedly decreased cell viability but the effect of all KIT inhibitors was significantly reduced in all three KIT mutant cell lines in the presence of FGF2 (Figure 3A). We evaluated the expression of the four FGF2 receptors in M230, HBL, and LND1 and showed that all three cell lines expressed FGFR2 and FGFR4 (Figure S2).

### 2.3. FGF2 Prevents BCL2 Reduction and BIM Induction to Inhibit Apoptosis

As we noticed that the cells treated with KIT inhibitors were dying, we analyzed the induction of apoptosis after treatment in these cells. We found that nilotinib induced the cleavage of PARP and caspase 7, which are markers of apoptosis, in M230, HBL, and LND1 cell lines (Figure 3B). To decipher the molecular mechanism of induction of apoptosis, we studied the effect of nilotinib on the different BCL2 family members (BCL2, MCL1, BID, BAK, BAD, BCL-XL, and BIM). Nilotinib induced a slight reduction of BCL2 and a strong induction of the BH3-only protein, BIM, in all three cell lines (Figure 3B). No variation in expression of the other BCL2 family members were observed (data not shown). Interestingly FGF2 prevented BCL2 reduction and BIM induction and drastically reduced the cleavage of PARP and caspase 7 (Figure 3B).

### 2.4. MEK Inhibition Reverses the FGF2-Mediated Resistance to KIT Inhibition

**Figure 4:**
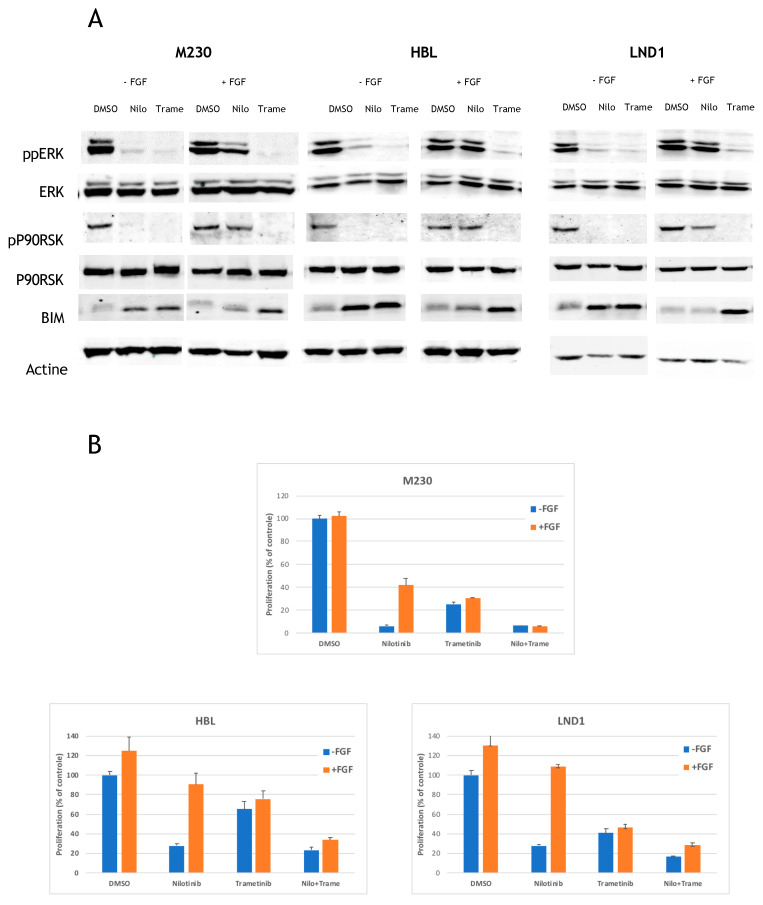
Effects of FGF2 MAPK pathway. (**A**) Cells were treated for 24 h with DMSO, 1 µM nilotinib or 0.2 µM Trametinib in the absence or in the presence of 20 ng/mL FGF2 and the levels of phosphorylated proteins or total proteins were analyzed by Western blotting. (**B**) Cells were treated with DMSO, 1 µM nilotinib, 0.2 µM trametinib or the combination of both in the absence or in the presence of 20 ng/mL FGF2 and proliferation was analyzed after 3 days (data are represented as mean +/− SD). The combination of nilotinib and trametinib significantly reduced proliferation in all three cell lines in the presence of FGF2 (M230, *p* < 0.0005; HBL, *p* < 0.01; LND1, *p* < 0.002; unpaired *t*-test).

To understand which signaling pathways were induced downstream of FGF2, we used a phospho-proteomics assay (R&D Systems, Minneapolis, MN, USA) to evaluate the activation of 43 kinases simultaneously in HBL cells in response to FGF2 and nilotinib. The assay confirmed the inhibition of the MAPK pathway (phosphorylation of ERK) and the PI3K pathway (phosphorylation of AKT and its substrates PRAS40, WNK1, and p27) by nilotinib, and demonstrated that FGF2 exclusively induced the MAPK pathway in this cell line (Figure S3). We confirmed these results in the three cell lines by Western blot using ERK and its substrate P90RSK as markers. Whereas nilotinib inhibits ERK and P90RSK phosphorylation, FGF2 prevented this inhibition (Figure 4A). Induction of BIM correlated with ERK/P90RSK inhibition (Figure 4A). To confirm the involvement of MAPK in regulating BIM in KIT-mutated melanoma, we used the MEK inhibitor trametinib. Trametinib inhibited ERK and P90RSK as expected and induced BIM expression even in the presence of FGF2 (Figure 4A). Next, we tested the effect of trametinib on M230, HBL, and LND1 proliferation in the presence and absence of nilotinib and FGF2. Trametinib only had a modest effect compared to nilotinib but its inhibition was insensitive to FGF2. Interestingly, the addition of trametinib efficiently reversed the FGF2-mediated resistance to nilotinib in each cell line (Figure 4B).

**Figure 5:**
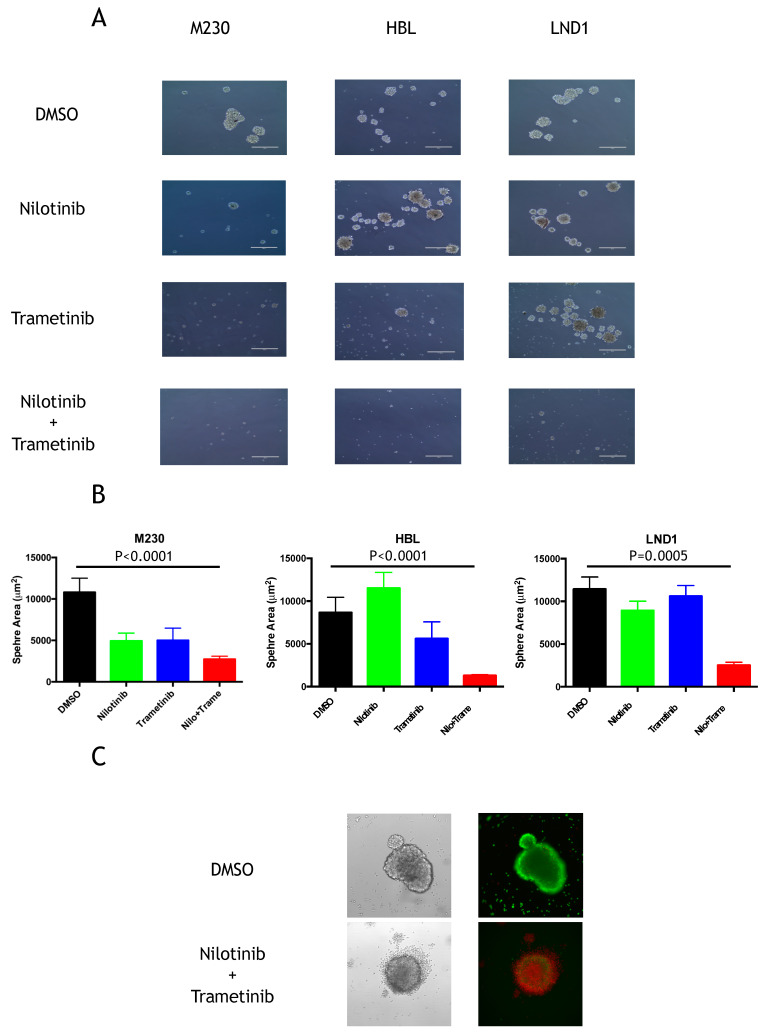
Effects of KIT and MAPK inhibitors on 3D proliferation. (**A**) Cells were dispensed in low-adherent plates in neural crest cell medium in the presence of DMSO, 1 µM nilotinib, 0.2 µM Trametinib or the combination of both and pictures were taken after 7 days (scale bar represents 400 µm). (**B**) Graphs represent mean of the sphere areas of around 30 spheres +/− SEM (similar results were obtained in two independent experiments). Variables were compared with the Mann–Whitney test. (**C**) HBL spheres formed after 7 days cells were treated with DMSO or the combination of 1 µM nilotinib and 0.2 µM Trametinib and after 48 h labelled for 1 h with the cell viability kit wherein living cells stain green and dead cells stain red. Magnification 20×. Representative of three independent experiments.

To confirm the importance of the MAPK pathway in a more physiological setting, we used cells grown as spheroids in a 3D model, which has proven to be a more representative model of the growth of tumors in vivo than cells grown as monolayers. M230, HBL, and LND1 were able to form large spheres in a neural crest cell medium and low adherence conditions. Interestingly, nilotinib had no inhibitory effect on the growth of HBL and LND1 spheres and only partially inhibited M230 sphere growth. Trametinib had no effect on HBL and only a modest inhibitory effect on M230 and LND1 growth. However, the combination of trametinib and nilotinib significantly reduced the number and the size of the spheres in all three cell lines (Figure 5A,B). We also treated HBL spheres once they were formed with the combination of trametinib and nilotinib for 48 h and tested their viability using a mixture of calcein-AM/ethidium bromide. We showed that the combination of both drugs induced strong cell death in spheroids compared to the control (Figure 5B).

As these data suggested the importance of the MAPK pathway in resistance to nilotinib, we analyzed its activation in situ in KIT-mutated melanoma. ERK and MEK phosphorylations were previously assessed using immunohistochemistry in eight samples from a phase II clinical trial with nilotinib in KIT-altered melanoma [16]. All tumors exhibited activation of both MEK and ERK at baseline, which was not significantly altered in response to nilotinib (Table S1). These observations suggested that, in KIT-mutated melanoma, the MAPK pathway may be activated independently of KIT in vivo.

## 3. Discussion

Despite the clear capacity of small-molecule inhibitors to target and inhibit KIT mutants in melanoma cell lines in culture, only infrequent tumor responses of typically minimal durability have been observed in clinical trials treating KIT-mutant melanoma patients with KIT inhibitors [10,15,16,17,18,19]. Moreover, in acral and mucosal melanoma, in which KIT alterations are frequent, only 20–30% of patients respond to anti-PD-1 immunotherapy [26]. In addition, the median progression-free survival after anti-PD-1 immunotherapy in these patients is reportedly short, approximately 4 months [27]. Finally, the efficacy of immunotherapies combining anti-PD1 and anti-CTLA4 remains lower in mucosal melanomas compared to cutaneous melanoma [27]. There is therefore a clear need for combination therapies to improve the prognosis for patients with KIT-mutated melanoma. To determine the profile of patients who could benefit from KIT inhibitors, we investigated the mechanisms of resistance to KIT inhibition therapy by exploring the mechanisms associated with response to nilotinib in different KIT-mutated melanoma cell lines. We extrapolated the results in melanoma patients using in situ data from a clinical trial. We used multiple inhibitors available in the clinics that share KIT as a target but have many nonoverlapping targets (e.g., dasatinib targets Src-family kinases). Each of the cell lines showed similar sensitivity to the different KIT inhibitors with the exception of imatinib in the HBL cell line carrying the D820YKIT mutant. This agrees with the experiment in Ba/F3 cells showing that mutations in the activation loop of KIT were resistant to imatinib [25], suggesting that melanoma patients with this type of mutation should be treated with second generation KIT inhibitors instead of imatinib. These data also agree with results from several clinical trials, which showed similar response rates with different KIT inhibitors (imatinib, nilotinib, dasatinib) [10,15,16,17,18,19]. The use of second or third generation KIT inhibitors cannot therefore circumvent the problem of resistance. A pharmacodynamic study during a phase II clinical trial with nilotinib in KIT-altered melanoma [16] showed a trend towards a reduction of growth factor expression in good responders compared to poor responders suggesting a link between growth factors and resistance to KIT inhibitors (Figure 2). These data suggest that resistance may be linked to coactivating pathways downstream of RTK rather than the clonal emergence of secondary mutations in the tumor. Indeed, the HGF-MET axis has been described as a mechanism of de novo resistance in a patient with a KIT-mutant melanoma [23]. Analysis of our patients’ tumors suggested that the FGF2 axis may also be a mechanism of resistance in KIT-mutant melanomas. Expression of FGF2 in melanoma is most likely due to a combination of autocrine secretion by tumoral cells and paracrine secretion by stromal cells from the tumor microenvironment. In good responders, the decrease in FGF2 expression in response to treatment could be explained by inhibition of signaling pathways in tumoral cells, causing FGF2 transcription to be reduced. For example, inhibition of the phosphorylation of STAT3 that we showed in good responders could play a role in the reduction of FGF2 expression [16]. In parallel a reduction of the tumor microenvironment in response to the treatment-induced tumor shrinkage will reduce the paracrine secretion of FGF2. Altogether these could explain the reduction of FGF2 in response to KIT inhibitors in good responders but need to be confirmed in our cellular system. The mechanisms described here could therefore be responsible for the transient response of good responders to therapy. As poor responders do not express high levels of FGF2, it is likely that FGF2 does not play a major role in de novo resistance to treatment. Using three melanoma cell lines which are dependent on oncogenic KIT, we showed that FGF2 drastically reduced the effect of the different KIT inhibitors pointing to an important role of the FGF2/FGFR axis in the resistance to KIT inhibitors. Although we did not directly show that blocking the FGF2/FGFR axis had an effect on tumor growth, it was recently shown that ponatinib, a dual KIT and FGFR inhibitor, caused much stronger inhibition of KIT-mutation-bearing melanoma in vivo than imatinib agreeing with our results [28]. We further show that this rescuing effect of FGF2 is due to the reactivation of the MAPK pathway. In accordance, in GISTs, inhibition of KIT by imatinib caused a feedback activation of FGF signaling and resulted in a rebound in ERK phosphorylation [29,30]. In our cell lines, the reactivation of the MAPK pathway inhibited expression of the pro-apoptotic protein BIM. BIM is one of the BCL-2-homology domain 3 only (BH3-only) proteins that share only the short BH3 domain with members of the BCL-2 family. BIM expression is strictly regulated by multi-site phosphorylation by several members of the MAP kinase group such as ERK and p90RSK. We showed, using a MEK inhibitor, that loss of MEK-ERK-RSK signaling leads to accumulation of BIM demonstrating that KIT and FGF2 control BIM expression through the MAPK pathway. BIM upregulation triggers cytochrome c release from mitochondria, which consequently induces a chain reaction that entails the formation of the apoptosome [31]. BIM has repeatedly emerged as a critical mediator of targeted therapy-induced apoptosis in multiple cancer types. In BRAF-mutant melanoma, BIM contributes to apoptosis induced by BRAF or MEK inhibitor treatment [32]. BIM has also been implicated in the apoptotic response of CML to imatinib [33]. BIM expression may therefore serve as a potential biomarker useful for predicting response to KIT inhibition in melanoma. Conversely, nilotinib also suppresses the STAT3 pathways that can lead to a reduction in BCL2 expression that acts in concert with BIM induction to trigger an apoptotic response and cell death in KIT-mutated melanoma. Activation of the MAPK pathway in KIT-mutated melanoma is seen in vivo in tumors, which exhibit activation of both MEK and ERK at baseline and upon treatment, suggesting that the MAPK pathway may be activated independently of KIT in these tumors. The importance of this pathway in contributing to resistance to KIT inhibitors was proved using MEK inhibitors alone or in combination with nilotinib. Indeed, although MEK inhibition only had a modest effect as a single agent, it efficiently reversed the FGF2-mediated resistance to nilotinib in each cell line. Moreover, we used a 3D model where cells grown as spheroids are resistant to nilotinib, making this model more representative of the growth of tumors in vivo than cells grown as monolayers. In this model, the combination of trametinib and nilotinib very efficiently inhibited the growth of the spheres, whereas single agents had no or only partial effect. Importantly, because the MAPK pathway is activated in both good and poor responders, MAPK pathway (re)activation is not the sole mechanism of resistance to KIT inhibitors and FGF2 is not the sole mediator of resistance.

## 4. Materials and Methods

### 4.1. Cell Culture and Reagents

Human melanoma cell line M230 was provided by Pr A. Ribas and was cultured in RPMI 1640 (Invitrogen, Cergy Pontoise, France) containing 10% (v/v) fetal calf serum (FCS; Perbio, Bredières, France), L-glutamin (2 mM; Gibco, Cergy pontoise, France), antibiotics (100 U/mL penicillin and 1000 μg/mL streptomycin; Gibco). Human melanoma cell lines HBL and LND1 were provided by Pr G E Gahnem and were cultured in F10 (Invitrogen) containing 10% (v/v) fetal calf serum (FCS; Perbio), L-glutamin (2 mM; Gibco), antibiotics (100 U/mL penicillin and 1000 μg/mL streptomycin; Gibco). Imatinib, nilotinib, sorafenib, dasatinib, pexidartinib, and trametinib were from Selleck Chemicals, dissolved in DMSO and used at 1 µM final concentration for KIT inhibitors and 0.2 µM for trametinib. FGF2 was from PeproTech and used at 20 ng/mL final concentration.

### 4.2. Three-Dimensional Spheroid Growth

Cells were plated at 2000 cells per mL density in DMEM/F12 medium supplemented with 1X B27, 10 ng/mL basic fibroblast growth factor, 20 ng/mL epidermal growth factor, and 5 ug/mL insulin in ultra-low attachment plates, treated three time a week with inhibitors or DMSO and sphere sizes were measured after 7 days. For short treatment assays, untreated spheres formed after 7 days were treated with DMSO 1 µM nilotinib or 0.2 µM Trametinib for 48 h and labelled with calcein-AM and ethidium bromide (Molecular Probes, Eugene, OR, USA) for 1 h at 37 °C according to the instruction of the manufacturer. After this time, pictures of the living cells (green) and dead cells (red) were taken using inverted fluorescence microscope.

### 4.3. Proliferation Assay

MTS assay: cells were dispensed into 96-well plates at 20,000 cell per well, in 180 µL of growing medium and 20 µL of inhibitor solution at the appropriate concentration with or without FGF2 (20 ng/mL) were added. After three days of treatment, proliferation was measured using 20 µL per well of CellTiter (Promega, Charbonnières, France).

### 4.4. Western Blotting

Melanoma cells were lysed in RIPA buffer (20 mM Tris-HCl pH 7.5, 150 mM NaCl, 1 mM Na2EDTA, 1 mM EGTA, 1% NP-40, 1% sodium deoxycholate, 2.5 mM sodium pyrophosphate, 1 mM beta-glycerophosphate, 1 mM Na3VO4) supplemented with proteinase inhibitor cocktail. Then, 20–25 µg of proteins were separated by SDS-PAGE and Western blot analysis was carried out according to standard protocols using the following antibodies (obtained from Cell Signaling Technology (Danvers, MA, USA) and used at 1:1000 dilution unless otherwise mentioned): phosphoKIT (Tyr719), KIT (C-19; Santa Cruz Biotechnology, Dallas, TX, USA), phosphoSTAT3 (Tyr705), STAT3 (F-2; Santa Cruz, Dallas, TX, USA), phosphoAKT XP (ser473), AKT, phosphoERK, ERK phosphoP90RSK (Thr359/Ser363), P90RSK, BAD, BAK, BAX, BCL2, BCL-XL, BID, BIM, FGFR1, FGFR2 (C-8; Santa Cruz Biotechnology, Dallas, TX, USA), FGFR3 (B-9; Santa Cruz Biotechnology, Dallas, TX, USA), FGFR4, and Actin (Abcam, Cambridge, UK). Proteins were revealed and quantified with SuperSignal^®^ West Pico Chemiluminescent Substrate (Thermo Scientific, Rockford, IL, USA) on an ImageQuant imaging system (GE Healthcare Bio-Science AB, Uppsala, Sweden) or by fluorescence on an Odyssey imaging system (LI-COR, Lincoln, NE, USA). Uncropped Western blot bands can be found in Figures S4–S6.

### 4.5. Immunofluorescence Staining

Four micrometer-thick frozen tumor sections were fixed in 3.7% formaldehyde for 20 min and permeabilized with 0.1% Triton X-100 in PBS for 10 min. Tumors sections were then incubated with a blocking solution composed of PBS containing 5% of FBS serum and 0.05% Tween20. Sections were then incubated with rabbit anti-Fibroblast Growth Factor-Basic (1–24) polyclonal antibody (#F3393, Sigma-Aldrich, Saint-Quentin Fallavier, France) overnight at 4 °C, washed three times with PBS-0.05% Tween20 and labeled with a Goat anti-rabbit IgG (H+L) Cross-Absorbed Secondary Antibody Alexa Fluor 568 conjugate (#A11011, Thermofisher Scientific, Cergy Pontoise, France) for 1 h. Samples were washed three times with PBS-0.05% Tween20, and then, coverslips were mounted with Vectashield Mounting Medium with Dapi (Vectors, Burlingame, CA, USA). Slides were observed with a confocal microscope (Zeiss LSM 800, Zeiss, Marly le Roi, France).

### 4.6. Flow Cytometry

After dissociation in PBS + 1 mM EDTA, 100,000 cells per condition were washed with PBS and incubated with IgG-PE (control) or CD117-PE (KIT) from Beckman Coulter. Cell were washed, suspended in PBS and analyzed using a CytoFlex flow cytometer (Beckman Coulter, Brea, CA, USA)

### 4.7. Pharmacodynamic Studies

Biopsies for pharmacodynamic analyses were performed for baseline, month 1, month 3, and month 6 if available. For each available sample, serial sections were used for nucleic acid extraction and immunostaining. Tumor cell content was assessed by a pathologist using hematoxylin–eosin staining. Tumor specimens were tested for mRNA expression of genes related to cell cycle, apoptosis and angiogenesis using a personalized qPCR array (SignArray, AnyGenes, France). Five 10 μm sections were extracted for RNA analysis from paraffin-embedded tissue specimens using RNeasy FFPE extraction kit (Qiagen, Courtabœuf, France) after xylene treatment according to the manufacturer’s protocol, or from frozen sections using RNeasy tissue extraction kit (Qiagen,). RNA quantity and quality were measured with the Nanodrop-ND-1000 (Nanodrop Technologies, Wilmington, NC, USA). First-strand cDNA was synthesized using a High-Capacity cDNA Archive Kit (Applied Biosystems, Courtabœuf, France) according to the manufacturer’s protocol. Transcript levels of genes related to cell cycle, apoptosis, and angiogenesis were measured using a personalized qPCR array (SignArray, AnyGenes, France). qRT-PCR was performed using Perfect Master Mix-Probe (AnyGenes, Paris, France) on LightCycler-480 (Roche Diagnostics, Meylan, France). Transcript levels were normalized to the housekeeping β-ACTIN and PPIA (peptidylprolyl isomerase A) transcripts. For the pharmacodynamic studies, continuous variables are summarized as mean ± SD. Variables were compared with the Mann–Whitney U test as appropriate. All statistical tests were two-sided. All statistical analyses were performed using Prism 6 (Graphpad Prism Software).

## 5. Conclusions

KIT-mutated melanoma, which represent around 22% of triple-wild-type melanoma, respond poorly to targeted therapies and immunotherapies. The study in this report indicates that the FGFR axis may be a mechanism of resistance in KIT-mutant melanomas through activation of the MAPK pathway. Therefore, the combined inhibition of KIT with FGFR or MEK may be a next-step effective clinical strategy in KIT-mutant melanoma.

## Supplementary Materials

The following are available online at https://www.mdpi.com/2072-6694/12/5/1062/s1, Figure S1: Expression of KIT at the membrane of melanoma cell lines, Figure S2: Expression of FGFR in melanoma cell lines, Figure S3: Phospho-arrays of HBL cells treated with nilotinib and FGF, Figures S4–S6: Uncropped Western blot bands, Table S1: Immunohistochemistry staining for phospho-MEK and phospho-ERK in tumors of patients treated with nilotinib, according to their best response following RECIST.

## References

[1] Wehrle-Haller B. (2003). The role of Kit-ligand in melanocyte development and epidermal homeostasis. Pigment. Cell Res..

[2] Lennartsson J., Jelacic T., Linnekin D., Shivakrupa R. (2005). Normal and oncogenic forms of the receptor tyrosine kinase kit. Stem Cells.

[3] Hemesath T.J., Price E.R., Takemoto C., Badalian T., Fisher D.E. (1998). MAP kinase links the transcription factor Microphthalmia to c-Kit signalling in melanocytes. Nature.

[4] Smalley K.S., Sondak V.K., Weber J.S. (2009). c-KIT signaling as the driving oncogenic event in sub-groups of melanomas. Histol. Histopathol..

[5] Cancer Genome Atlas N. (2015). Genomic Classification of Cutaneous Melanoma. Cell.

[6] Curtin J.A., Busam K., Pinkel D., Bastian B.C. (2006). Somatic activation of KIT in distinct subtypes of melanoma. J. Clin. Oncol..

[7] Bastian B.C., Esteve-Puig R. (2013). Targeting activated KIT signaling for melanoma therapy. J. Clin. Oncol..

[8] Gong H.Z., Zheng H.Y., Li J. (2018). The clinical significance of KIT mutations in melanoma: A meta-analysis. Melanoma Res..

[9] Dumaz N., Andre J., Sadoux A., Laugier F., Podgorniak M.P., Mourah S., Lebbe C. (2015). Driver KIT mutations in melanoma cluster in four hotspots. Melanoma Res..

[10] Jiang X., Zhou J., Yuen N.K., Corless C.L., Heinrich M.C., Fletcher J.A., Demetri G.D., Widlund H.R., Fisher D.E., Hodi F.S. (2008). Imatinib targeting of KIT-mutant oncoprotein in melanoma. Clin. Cancer Res..

[11] Monsel G., Ortonne N., Bagot M., Bensussan A., Dumaz N. (2010). c-Kit mutants require hypoxia-inducible factor 1alpha to transform melanocytes. Oncogene.

[12] Smalley K.S., Contractor R., Nguyen T.K., Xiao M., Edwards R., Muthusamy V., King A.J., Flaherty K.T., Bosenberg M., Herlyn M. (2008). Identification of a novel subgroup of melanomas with KIT/cyclin-dependent kinase-4 overexpression. Cancer Res..

[13] Meng D., Carvajal R.D. (2019). KIT as an Oncogenic Driver in Melanoma: An Update on Clinical Development. Am. J. Clin. Dermatol..

[14] Blanke C.D., Demetri G.D., von Mehren M., Heinrich M.C., Eisenberg B., Fletcher J.A., Corless C.L., Fletcher C.D., Roberts P.J., Heinz D. (2008). Long-term results from a randomized phase II trial of standard-versus higher-dose imatinib mesylate for patients with unresectable or metastatic gastrointestinal stromal tumors expressing KIT. J. Clin. Oncol..

[15] Cho J.H., Kim K.M., Kwon M., Kim J.H., Lee J. (2012). Nilotinib in patients with metastatic melanoma harboring KIT gene aberration. Investig. New Drugs.

[16] Delyon J., Chevret S., Jouary T., Dalac S., Dalle S., Guillot B., Arnault J.P., Avril M.F., Bedane C., Bens G. (2018). STAT3 Mediates Nilotinib Response in KIT-Altered Melanoma: A Phase II Multicenter Trial of the French Skin Cancer Network. J. Investig. Derm..

[17] Guo J., Si L., Kong Y., Flaherty K.T., Xu X., Zhu Y., Corless C.L., Li L., Li H., Sheng X. (2011). Phase II, open-label, single-arm trial of imatinib mesylate in patients with metastatic melanoma harboring c-Kit mutation or amplification. J. Clin. Oncol..

[18] Hodi F.S., Corless C.L., Giobbie-Hurder A., Fletcher J.A., Zhu M., Marino-Enriquez A., Friedlander P., Gonzalez R., Weber J.S., Gajewski T.F. (2013). Imatinib for Melanomas Harboring Mutationally Activated or Amplified KIT Arising on Mucosal, Acral, and Chronically Sun-Damaged Skin. J. Clin. Oncol..

[19] Minor D.R., Kashani-Sabet M., Garrido M., O’Day S.J., Hamid O., Bastian B.C. (2012). Sunitinib therapy for melanoma patients with KIT mutations. Clin. Cancer Res..

[20] Lee J.H., Kim Y., Choi J.W., Kim Y.S. (2013). Correlation of imatinib resistance with the mutational status of KIT and PDGFRA genes in gastrointestinal stromal tumors: A meta-analysis. J. Gastrointest. Liver Dis..

[21] Si L., Xu X., Kong Y., Flaherty K.T., Chi Z., Cui C., Sheng X., Li S., Dai J., Yu W. (2012). Major response to everolimus in melanoma with acquired imatinib resistance. J. Clin. Oncol..

[22] Cho J., Kim S.Y., Kim Y.J., Sim M.H., Kim S.T., Kim N.K.D., Kim K., Park W., Kim J.H., Jang K.T. (2017). Emergence of CTNNB1 mutation at acquired resistance to KIT inhibitor in metastatic melanoma. Clin. Transl. Oncol..

[23] Oba J., Kim S.H., Wang W.L., Macedo M.P., Carapeto F., McKean M.A., Van Arnam J., Eterovic A.K., Sen S., Kale C.R. (2018). Targeting the HGF/MET Axis Counters Primary Resistance to KIT Inhibition in KIT-Mutant Melanoma. JCO Precis. Oncol..

[24] Chraybi M., Abd Alsamad I., Copie-Bergman C., Baia M., André J., Dumaz N., Ortonne N. (2013). Oncogene abnormalities in a series of primary melanomas of the sinonasal tract: NRAS mutations and CCND1 amplification are more frequent than KIT or BRAF mutations. Human Pathol..

[25] Guo J., Carvajal R.D., Dummer R., Hauschild A., Daud A., Bastian B.C., Markovic S.N., Queirolo P., Arance A., Berking C. (2017). Efficacy and safety of nilotinib in patients with KIT-mutated metastatic or inoperable melanoma: Final results from the global, single-arm, phase II TEAM trial. Ann. Oncol..

[26] Shoushtari A.N., Munhoz R.R., Kuk D., Ott P.A., Johnson D.B., Tsai K.K., Rapisuwon S., Eroglu Z., Sullivan R.J., Luke J.J. (2016). The efficacy of anti-PD-1 agents in acral and mucosal melanoma. Cancer.

[27] D’Angelo S.P., Larkin J., Sosman J.A., Lebbe C., Brady B., Neyns B., Schmidt H., Hassel J.C., Hodi F.S., Lorigan P. (2017). Efficacy and Safety of Nivolumab Alone or in Combination With Ipilimumab in Patients With Mucosal Melanoma: A Pooled Analysis. J. Clin. Oncol..

[28] Han Y., Gu Z., Wu J., Huang X., Zhou R., Shi C., Tao W., Wang L., Wang Y., Zhou G. (2019). Repurposing Ponatinib as a Potent Agent against KIT Mutant Melanomas. Theranostics.

[29] Li F., Huynh H., Li X., Ruddy D.A., Wang Y., Ong R., Chow P., Qiu S., Tam A., Rakiec D.P. (2015). FGFR-Mediated Reactivation of MAPK Signaling Attenuates Antitumor Effects of Imatinib in Gastrointestinal Stromal Tumors. Cancer Discov..

[30] Javidi-Sharifi N., Traer E., Martinez J., Gupta A., Taguchi T., Dunlap J., Heinrich M.C., Corless C.L., Rubin B.P., Druker B.J. (2015). Crosstalk between KIT and FGFR3 Promotes Gastrointestinal Stromal Tumor Cell Growth and Drug Resistance. Cancer Res..

[31] Hata A.N., Engelman J.A., Faber A.C. (2015). The BCL2 Family: Key Mediators of the Apoptotic Response to Targeted Anticancer Therapeutics. Cancer Discov..

[32] Cartlidge R.A., Thomas G.R., Cagnol S., Jong K.A., Molton S.A., Finch A.J., McMahon M. (2008). Oncogenic BRAF(V600E) inhibits BIM expression to promote melanoma cell survival. Pigment. Cell Melanoma Res..

[33] Belloc F., Moreau-Gaudry F., Uhalde M., Cazalis L., Jeanneteau M., Lacombe F., Praloran V., Mahon F.X. (2007). Imatinib and nilotinib induce apoptosis of chronic myeloid leukemia cells through a Bim-dependant pathway modulated by cytokines. Cancer Biol..

