# Supplementary material for: FGF2 Induces Resistance to Nilotinib through MAPK Pathway Activation in KIT Mutated Melanoma
Source: Cancers (Basel). 2020 Apr 25;12(5):1062. doi: 10.3390/cancers12051062 (PMC7281633; doi:10.3390/cancers12051062)
Supplement: Supplementary file 1 [file cancers-12-01062-s001.zip › cancers-743443-supplementary figures.pdf]

Supplementary Material

FGF2 Induces Resistance to Nilotinib through MAPK Pathway Activation in KIT Mutated Melanoma

Pauline Tétu, Julie Delyon, Jocelyne André, Coralie Reger de Moura, Malak Sabbah, Ghanem E Ghanem, Maxime Batistella, Samia Mourah, Céleste Lebbé and Nicolas Dumaz

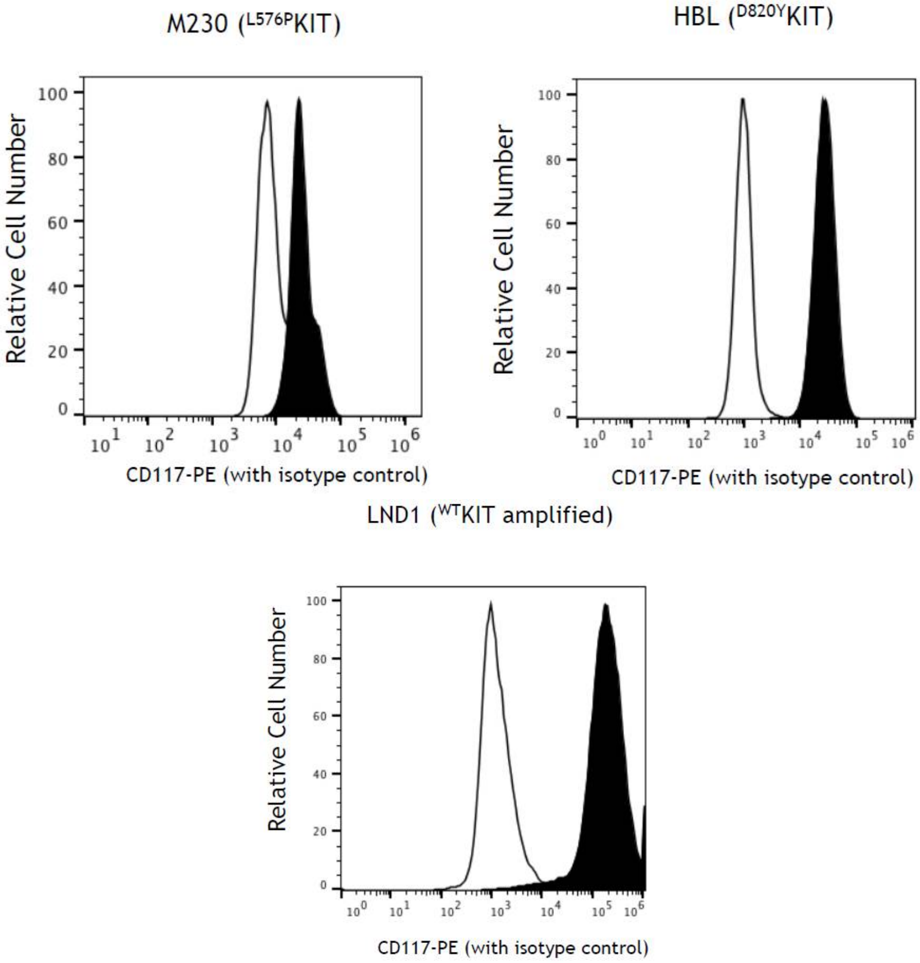

Figure S1. Expression of KIT at the membrane of melanoma cell lines.

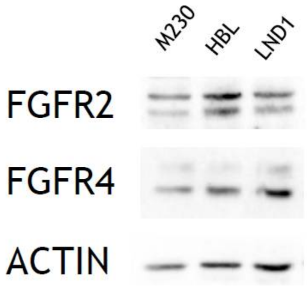

Figure S2. Expression of FGFR in melanoma cell lines.

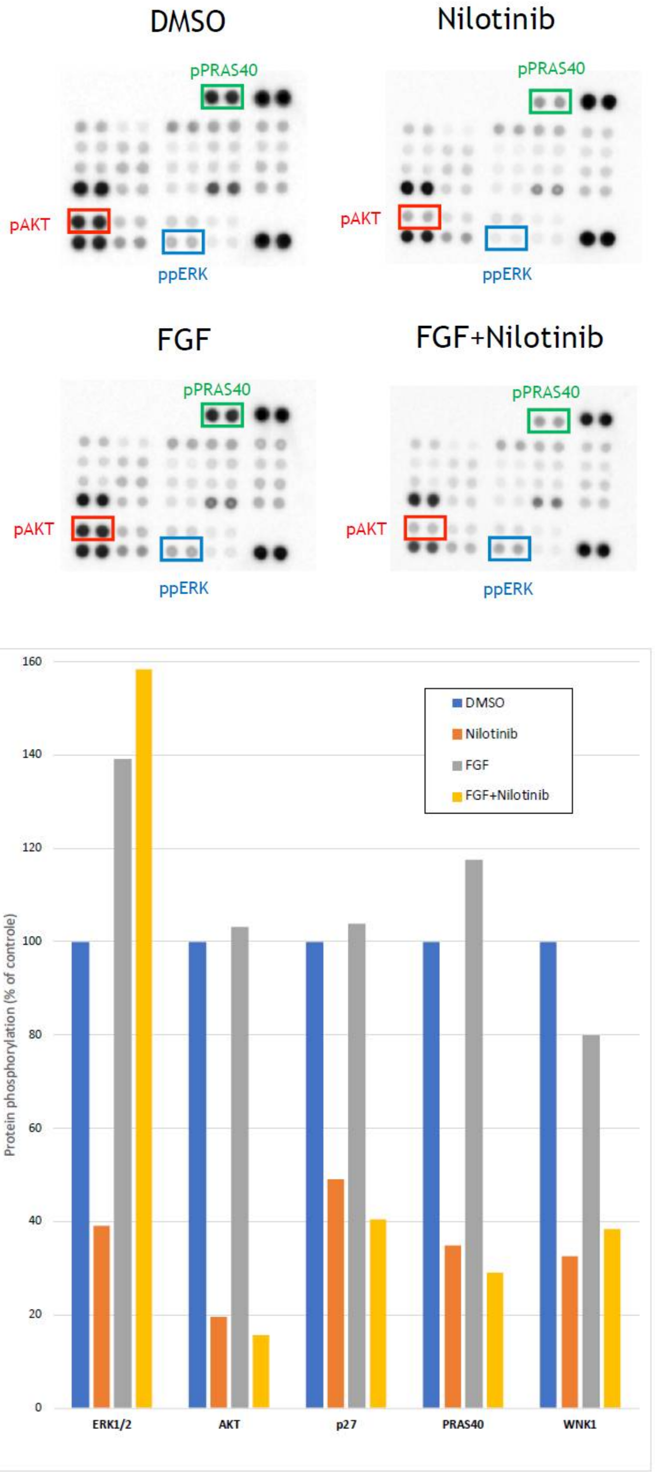

Figure S3. Phospho-arrays of HBL cells treated with nilotinib and FGF.

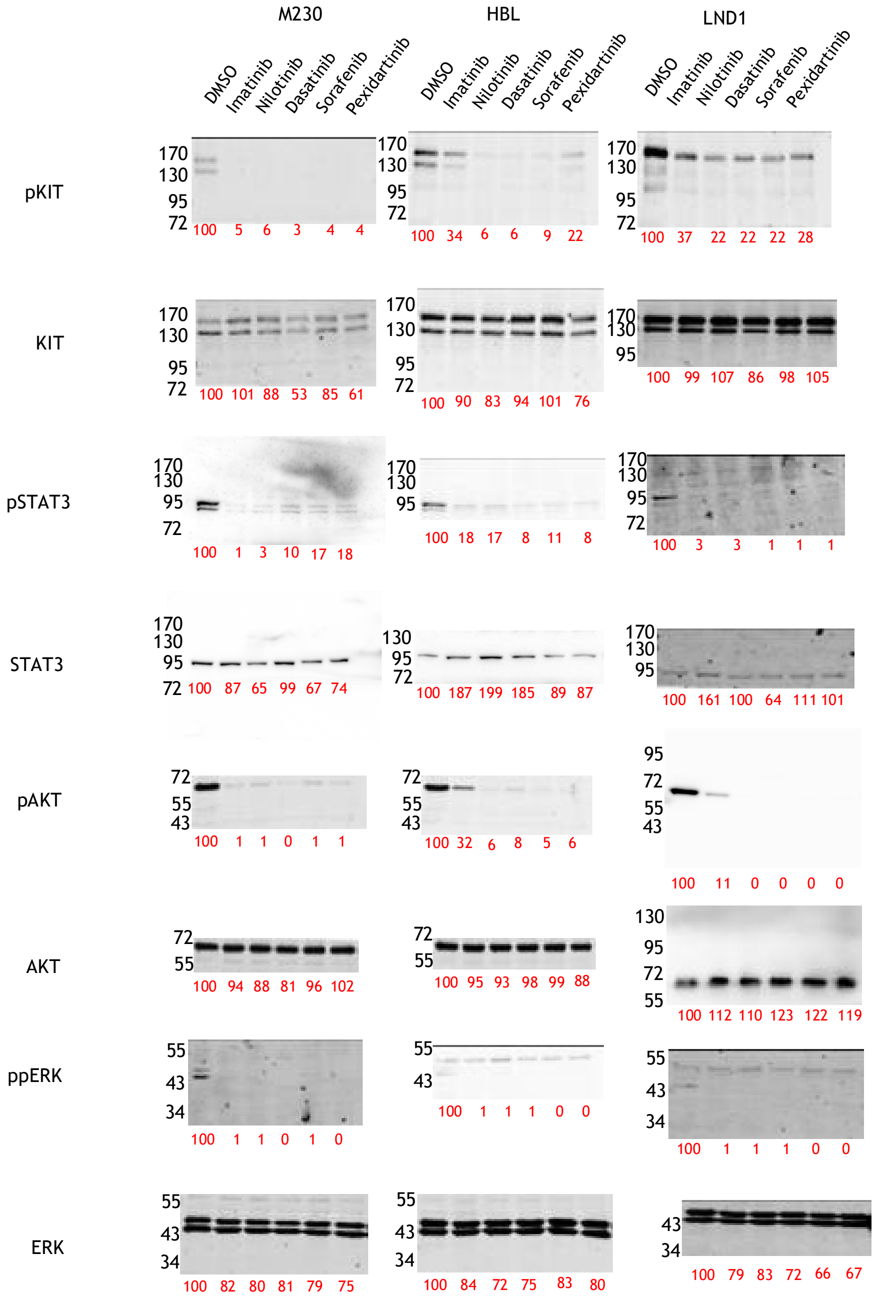

Figure S4. Uncropped Western blot bands for Figure 1. Cells were treated for 24 hrs with DMSO or 1uM inhibitors and the levels of phosphorylated proteins or total proteins were analyzed by Western Blotting. Quantification of western blots by ImageJ is indicated in red below each blot as percentage of DMSO.

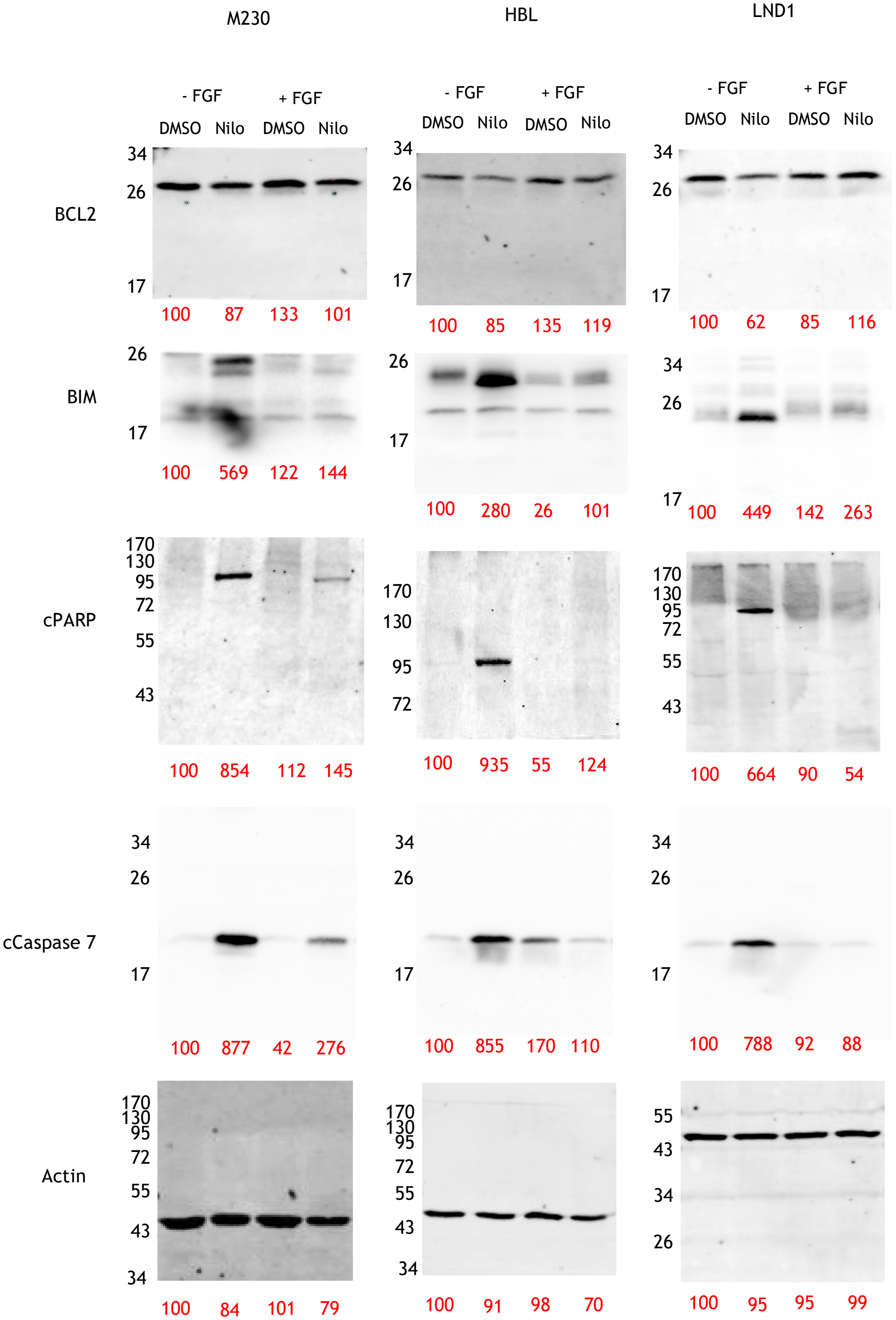

Figure S5. Uncropped Western blot bands for Figure 3. Cells were treated for 24 hrs with DMSO or 1uM nilotinib in the absence or in the presence of 20 ng/ml FGF2 and the levels of cleaved proteins

(cPARP and cCaspase 7) or total protein were analyzed by Western Blotting. Quantification of western blots by ImageJ is indicated in red below each blot as percentage of DMSO.

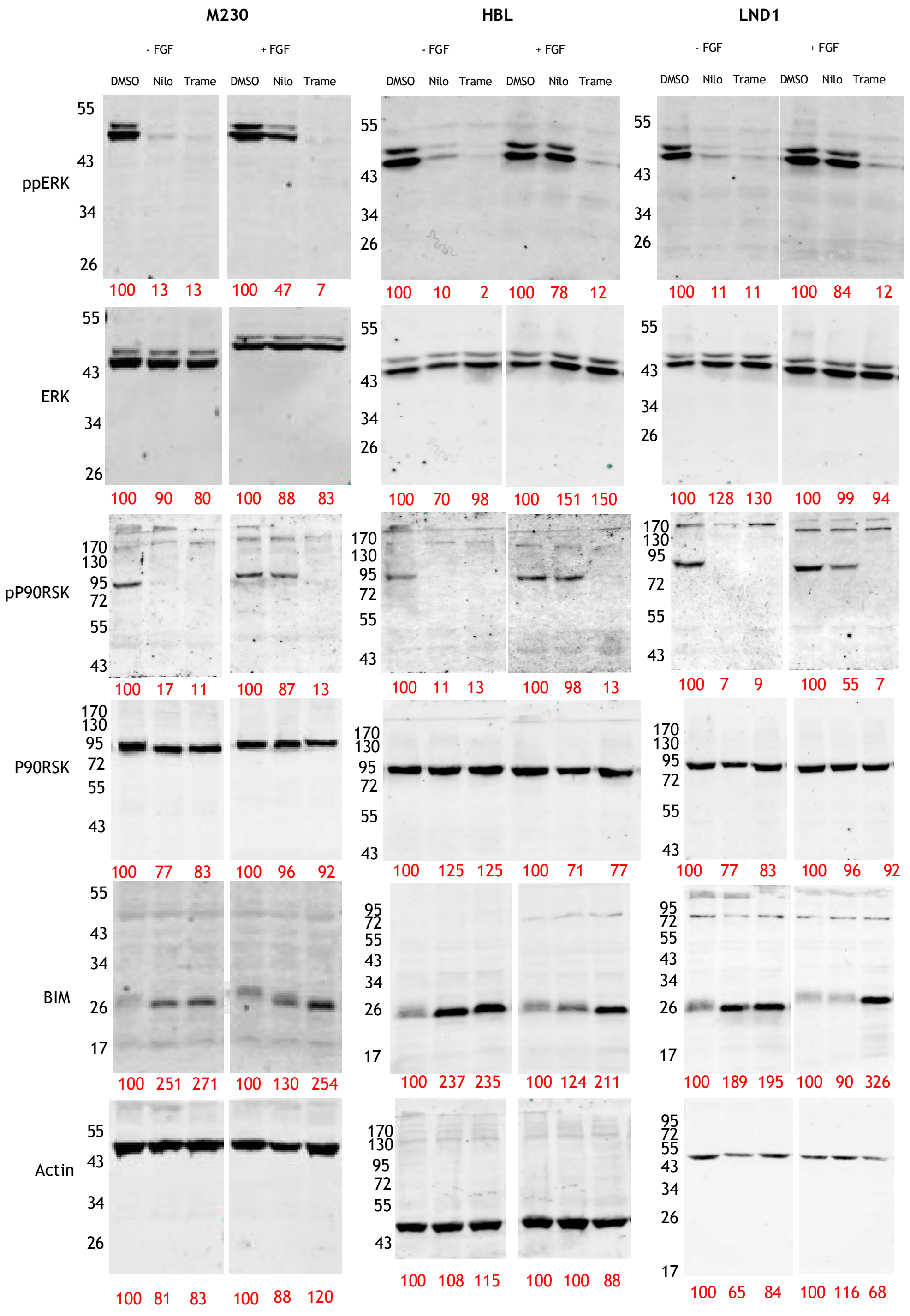

Figure S6. Uncropped Western blot bands for Figure 4. Cells were treated for 24 hrs with DMSO, 1uM nilotinib or 0.2uM Trametinib in the absence or in the presence of 20 ng/ml FGF2 and the levels of phosphorylated proteins or total proteins were analyzed by Western Blotting.

© 2019 by the authors. Licensee MDPI, Basel, Switzerland. This article is an open access article distributed under the terms and conditions of the Creative Commons Attribution (CC BY) license (http://creativecommons.org/licenses/by/4.0/).
