# Supplementary material for: FGF2 Induces Resistance to Nilotinib through MAPK Pathway Activation in KIT Mutated Melanoma
Source: Cancers (Basel). 2020 Apr 25;12(5):1062. doi: 10.3390/cancers12051062 (PMC7281633; doi:10.3390/cancers12051062)
Supplement: Supplementary file 1 [file cancers-12-01062-s001.zip › cancers-743443-supplementary Table S1.pdf]

Table S1 : Immunohistochemistry staining for phospho-MEK and phospho-ERK in tumors of patients treated with nilotinib, according to their best response following RECIST.

		**Patients with PD or SD< 3 months as best response**			**Patients with CR, PR or SD> 3 months as best response**			
		**#1**<br>^{R634L}**KIT**	**# 2**<br>^{L576P}**KIT**	**# 3**<br>^{K642E}**KIT**	**#A**<br>^{L576R}**KIT**	**#B**<br>^{AMP}**KIT**	**#C**<br>^{G565V}**KIT**	**#D**<br>^{K642E}**KIT**
**pMEK**	**Baseline**<br>- Tumor cell (%)<br>- Staining	100<br>++	40<br>+	0<br>NA	100<br>+++	80<br>+	70<br>+	100<br>+++
	**M1**<br>- Tumor cell (%)<br>- Staining	100<br>++	20<br>+	80<br>++	100<br>+++	50<br>+	60<br>+	100<br>+++
	**M6**<br>- Tumor cell (%)<br>- Staining	NA	NA	90<br>++	100<br>+++	NA	NA	100<br>+++
**ppERK**	**Baseline**<br>- Tumor cell (%)<br>- Staining	100<br>++	30<br>+	0<br>NA	100<br>++	80<br>+	80<br>+	100<br>+
	**M1**<br>- Tumor cell (%)<br>- Staining	100<br>+++	40<br>+	20<br>+	50<br>+	10<br>+	80<br>+	50<br>++
	**M6**<br>- Tumor cell (%)<br>- Staining	NA	NA	100<br>++	100<br>++	60<br>+	60<br>+	80<br>++

Phospho-MEK and phospho-ERK stainings were quantified using a semi-quantitative scale: 0, no staining; +, weakly positive; ++, moderately positive; +++, strongly positive staining. NA, not available.
